# Aging, Estrogen Loss and Epoxyeicosatrienoic Acids (EETs)

**DOI:** 10.1371/journal.pone.0070719

**Published:** 2013-08-13

**Authors:** Alison R. Lee, Angela S. Pechenino, Hua Dong, Bruce D. Hammock, Anne A. Knowlton

**Affiliations:** 1 Molecular & Cellular Cardiology, Cardiovascular Division, Department of Medicine, University of California Davis, Davis, California, United States of America; 2 Department of Pharmacology, University of California Davis, Davis, California, United States of America; 3 Department of Entymology, University of California Davis, Davis, California, United States of America; 4 The Department of Veteran's Affairs, Northern California VA, Sacramento, California, United States of America; Indiana University, United States of America

## Abstract

Inflammation is a key element in many cardiovascular diseases. Both estrogen loss, caused by menopause, and aging have inflammatory consequences. Epoxyeicosatrienoic acids (EETs) are anti-inflammatory molecules synthesized by various cytochrome P450 (Cyp) enzymes from arachidonic acid. EETs are in the third (Cytochrome P450) pathway of arachindonic acid metabolism, others being cyclooxygenases and lipoxygenases. We hypothesized that aging and estrogen loss would reduce levels of anti-inflammatory EETs. Adult (6 mo) and aged (22 mo) ovariectomized rats with (OP) and without (Ovx) 17-∃-estradiol replacement were used in this study. Mass spectrometry was used to measure levels of EETs and their metabolites, dihydroxyeicosatrienoic acids (DHETs). Levels of Cyp2C2, Cyp2C6, and Cyp2J2, the principal Cyps responsible for EETs synthesis, as well as soluble epoxide hydrolase (sEH), which metabolizes EETS to DHETs, were determined via western blot. Overall Cyp levels decreased with age, though Cyp2C6 increased in the liver. sEH was increased in the kidney with estrogen replacement. Despite protein changes, no differences were measured in plasma or aortic tissue levels of EETs. However, plasma 14,15 DHET was increased in aged Ovx, and 5,6 DHET in adult OP. In conclusion neither aging nor estrogen loss decreased the anti-inflammatory EETs in the cardiovascular system.

## Introduction

Menopause is characterized by dramatically decreased estrogen levels and a marked acceleration of atherosclerosis and heart disease in women [1]. Although a loss of estrogen is blamed for these effects and a number of animal studies support this [2], [3], clinical studies showed no benefit from hormone replacement treatment(HRT) for post-menopausal females [4], [5]. In fact, although nonrandomized studies had supported that HRT was cardio-protective for women post-menopause, the randomized, double-blinded clinical trials showed an increase in cardiovascular events, particularly during the first year of treatment. Further analysis of these results led to the timing hypothesis [6], that a long delay between menopause and HRT was a factor in the increased cardiovascular disease in the trials. Recent work from our lab indicates that delayed estrogen administration leads to an increase in inflammatory gene expression [7], signifying that inflammation plays a role in the deleterious effects seen with late estrogen administration in the clinical trials. Because inflammatory changes have been definitively associated with the development of atherosclerosis [8], [9], the identification of antioxidant and anti-inflammatory pathways and molecules has become paramount.

**Figure 1:**
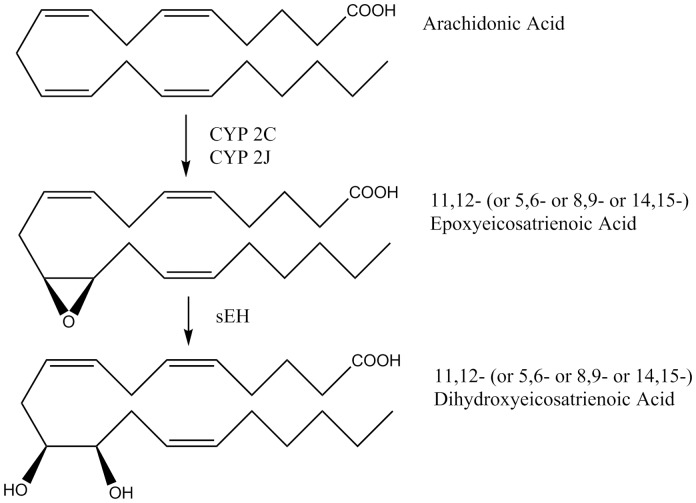
Diagram summarizes key steps in EETs synthesis and metabolism.

There are three arachidonic acid metabolism pathways: the COX (cycloxygenase) pathway, the LOX (lipoxygenase) pathway and the CYP (cytochrome P450) pathway. The importance of the first two pathways, which produce prostaglandins and leukotrienes, is well-established in the cardiovascular system; however, the role of the CYP pathway, which produces epoxyecosatrienoic acids (EETs), has yet to be fully characterized. EETs, of which there are 4 isoforms (5,6-, 8,9-, 11,12-, and 14,15-), are lipid signaling molecules synthesized primarily by the action of 2 families of cytochrome P450 (Cyp) enzymes, Cyp2C and Cyp2J, on arachadonic acid [10]. Recent evidence indicates that EETs act as important anti-inflammatory and antioxidant molecules [10], [11]. EETs have been shown to protect against ischemic injury by reducing inflammation [12], and have important stimulatory effects on angiogenesis [13]. EETs are predominantly metabolized by soluble epoxide hydrolase (sEH) into their corresponding dihydroxyeicosatrienoic acid (DHET) isoforms (Figure 1). While other epoxide hydrolases are of little importance in EET metabolism, other metabolic pathways, such as ∃-oxidation, T-oxidation and chain elongation, are of some importance, particularly when sEH is suppressed [14]–[16]. sEH polymorphisms are associated with an increased risk in atherosclerosis in a number of clinical studies [17]–[19], and treatment of apolipoprotein E knockout mice with synthesized sEH inhibitors for 8 weeks reduced atherosclerosis in these animals [20]. The function of DHETs is less clear, as early studies showed injurious effects, but later ones are not definitive on this issue [21], [22]. Because a number of potential therapeutic agents have been developed that inhibit sEH, we were interested if aging and estrogen loss, which are associated with increased inflammation, alter the expression of EETs or the enzymes involved in their synthesis and degradation. Inhibition of sEH to increase expression of the anti-inflammatory EETs has the potential to be a therapeutic approach to ameliorate some of the adverse changes of aging.

## Materials and Methods

### Animal Model

Norway-Brown rats , 4–6 months [Adult] and 19–22 months [Aged] of aged, were obtained from the National Institutes on Aging (Bethesda, MD), housed in standard female only conditions, and fed standard laboratory rat chow. All rats underwent ovariectomy (ovx) and half received 17∃-estradiol (E2) replacement using a 0.5 mg E2 sustained release capsule (Innovative Research, Sarasota, FL) implanted at the time of ovx, as previously described [7], [23]. Rats were divided into 4 groups (Adult Ovx, Adult ovx with immediate estrogen replacement [Adult OP], Aged Ovx, and Aged ovx with immediate estrogen replacement [Aged OP]). Tissue collection was done 9 weeks post ovariectomy based on our findings that following ovariectomy there appears to be a cascade of changes over at least a 9 week period, and that this amount of time is required for cardiac levels of heat shock protein 72 to decline to levels seen in males [24]. All samples were collected at the same time of day. Plasma samples and the liver, kidney, left ventricle, aorta and uterus were collected at 9 weeks, flushed with ice-cold PBS and flash frozen in liquid nitrogen. At the time of tissue collection, the uterus was weighed to verify treatment groups. All animal protocols were approved by the University of California, Davis Animal Research Committee in accordance with the National Institutes of Health *Guide for the Care and Use of Laboratory Animals*.

### Liquid Chromatography/Mass Spectrometry for oxylipin profiling analysis - Plasma sample preparation

Plasma samples were spiked with 10: L 500 nM internal standard I (d4-6-keto-PGF1a, d4-PGE2, d4-TXB2, d4-LTB4, d11-14,15-DiHETrE, d6-20-HETE, d4-9-HODE, d8-12-HETE, d8-5-HETE, d11-11(12)-EpETrE, d4-9(10)-EpOME, d8-AA) and then were extracted by solid phase extraction using Oasis HLB cartridges (3cc 60mg, Waters, Milford, MA). The HLB cartridges were first washed with 2 mL ethyl acetate, 2 mL methanol twice, and 2 mL 95∶5 v/v water/methanol with 0.1% acetic acid. The 6 mL heart perfusate samples were then loaded in duplicates onto the cartridges with 3 mL samples per cartridge. 10 :L of butylated hydroxyl toluene (BHT) was added to each sample after loading. The samples were then washed with 6 mL 95∶5 v/v water/methanol with 0.1% acetic acid and dried for 20 min with low vacuum. The target analytes were then eluted with 0.5 mL methanol followed by 2 mL of ethyl acetate into the tubes with 6 :L 30% glycerol in methanol as the trap solution. The volatile solvents were evaporated by using vacuum centrifugation (Speed-Vac) until 2 :L trap solution remained in the tube. The residues were dissolved in 50 :L of methanol containing 200 nM internal standards II (1-cyclohexyl-dodecanoic acid urea, CUDA). The samples were mixed with a vortex mixer for 2 min, centrifuged at 14000 x g for 5 min and then transferred to auto sampler vials with 150 :L inserts for LC/MS/MS analysis.

### Aorta sample preparation

Aortic tissues (25–50 mg) were collected, flash-frozen in liquid nitrogen and stored at −20°C for extraction. After weighing, the aorta samples were spiked with 10 :L 500 nM internal standard I as described above. 400 :L of ice-cold methanol with 0.1 % of acetic acid and 0.1% of BHT were added onto tissue samples and samples were incubated at −20°C for 30 min. Samples were then homogenized at 30 Hz for 30 min and stored at −20°C freezer overnight. The supernatants were collected after centrifugation at 10,000 rpm for 10 min. The remaining pellets were washed with 100 :L of ice-cold methanol with 0.1 % of acetic acid and 0.1% of BHT and centrifuged. The supernatants of each sample were combined and diluted with 2 mL of H_2_O and load onto SPE cartridges. Further sample preparation was as described for plasma sample preparation.

### LC/MS/MS Analysis

Liquid chromatography/tandem MS (LC/MS/MS) analysis of oxylipins was performed using a modified method based on the previous publication [25]. An Agilent 1200 SL liquid chromatography series (Agilent Corporation, Palo Alto, CA) with an Agilent Eclipse Plus C18 2.1 x 150 mm, 1.8 :m column was used for the oxylipins separation. The mobile phase A was water with 0.1% acetic acid while the mobile phase B was composed of acetonitrile/methanol (80/15, v/v) and 0.1% acetic acid. Gradient elution was performed at a flow rate of 250 :L/min and the gradient used is described in Table 1. The injection volume was 10 :L and the samples were kept at 4°C in the auto sampler. Analytes were detected by negative MRM mode using a 4000 QTrap tandem mass spectrometer (Applied Biosystems Instrument Corporation, Foster City, CA) equipped with an electrospray ionization source (Turbo V). The QTrap was set as follows: CUR  = 20 psi, TEM  = 500°C, GS1  = 50 psi, GS2  = 30 psi, CAD  =  High, IS  = −4500 V, DP  = −60 V, EP  = −10 V. Calibration curves were generated by 10 :L injections of seven standards containing each analyte, internal standard I, and internal standard II for quantification purpose. The EETs levels represent free EETs.

**Table 1:** LC Mobile Phase Gradient.

Total Time	Flow Rate	A (%)	B (%)
(min)	(:L/min)		
0	250	65	35
0.25	250	65	35
1	250	55	45
3	250	45	55
8.5	250	34	66
12.5	250	28	72
15	250	18	82
16.5	250	5	95
18	250	5	95
18.1	250	65	35
21.5	250	65	35

Details of elution gradient.

### Western blot analysis

Analysis was performed as previously described [26]. Prior to analysis, albumin was removed from the liver samples, as the abundant amount of this protein interfered with analysis of other proteins of similar size. Samples from a 3∶1 mixture with Affi-gel blue (Bio-Rad, Hercules, CA) were agitated for 30 minutes at 4° C and briefly centrifuged to remove the Affi-gel blue which is cross-linked to agarose beads. The supernatant was used for analysis of liver protein expression. All other tissues were processed in a standard manner as previously described [26]. Antibodies were used in the following dilutions: Cyp2C2 (Santa Cruz, Santa Cruz, CA) 1∶1000; Cyp2C6 (Santa Cruz) 1∶1000; Cyp2J2 (Santa Cruz) 1∶1000; sEH (Cayman Chemicals, Ann Arbor, MI) 1∶1000. The appropriate HRP-conjugated secondary antibodies of anti-mouse or anti-rabbit (GE, UK) were used at 1∶1000 dilutions and developed using West Pico enhanced chemiluminescence (Thermo Scientific, Waltham, MA). Proteins were normalized to GAPDH, which did not vary among groups. When separate gels were run for multiple samples, internal normalization controls were used in order to accurately compare the gels.

### Data analysis

Results are presented as the mean +/− SEM of at least three separate experiments. Data were analyzed by a one-way ANOVA or an ANOVA on Ranks, followed by a Student Neuman Keuls test or Dunn's test, where appropriate. A p < 0.05 was considered significant (Sigma Stat).

## Results

**Figure 2:**
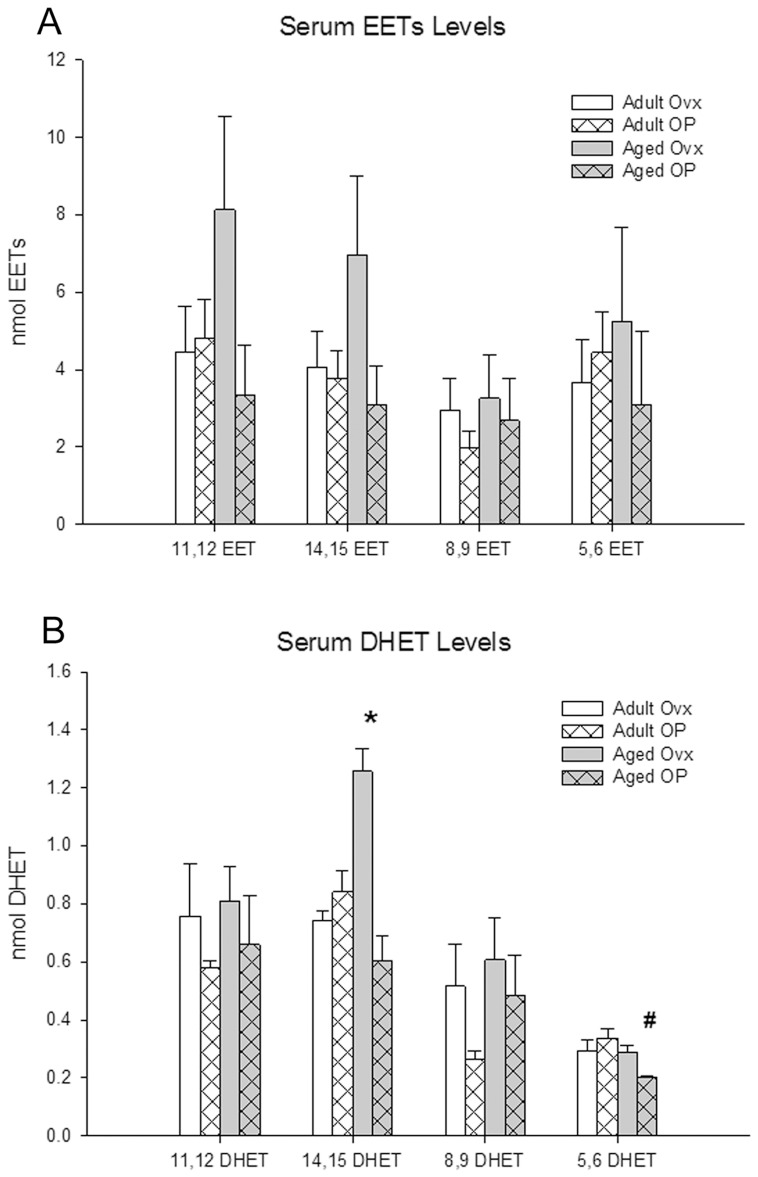
Plasma levels of EETs and DHETs from MS analysis. A: EETs levels. B: DHETs levels. Adult Ovx- white bars, Adult OP- white bars with pattern, Aged Ovx- grey bars, Aged OP- grey bars with pattern. # P<0.05 compared with Adult OP, * P<0.001 compared with all. n = 6–8/group for EETs; n = 5–8/group for DHETs, except 5,6 DHET where n = 3–4/group.

Plasma EETs levels (the four isomers) did not differ based on either age or estrogen status (Fig. 2A). In contrast, the DHETs showed some marked differences (Fig. 2B). The 14,15-DHET levels in the Aged Ovx group were higher than the other treatment groups. The 5,6-DHETs in the aged OP group were lower than those measured in the adult OP group. The plasma 11,12- and 8,9-DHET levels did not differ among the groups.

**Figure 3:**
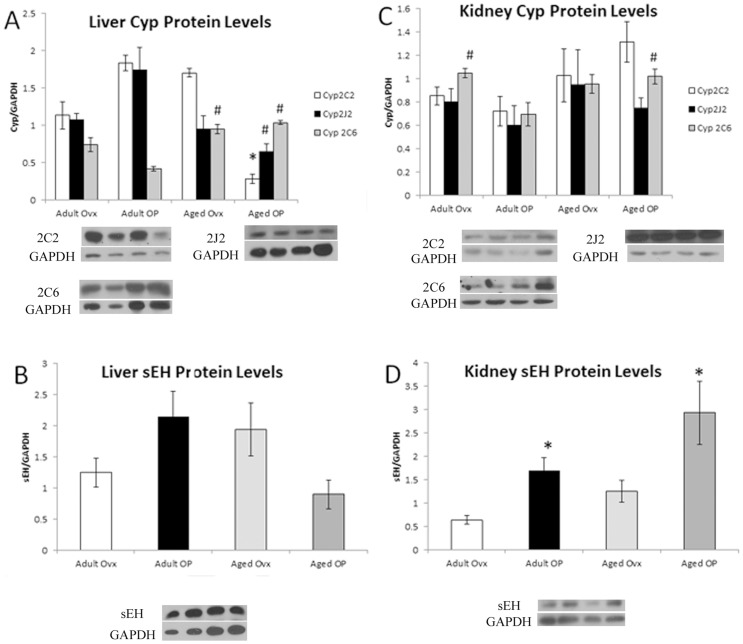
Western blot analysis of liver and kidney EETs related genes, normalized to GAPDH. A representative Western blot showing bands from each of the 4 groups in the order they are given on the graph plus a representative GAPDH blot are shown for each graph. A: Cyp2C2, Cyp2J2 and Cyp2C6 in liver. B: sEH in liver. C: Cyp2C2, Cyp2J2 and Cyp2C6 in kidney. D: sEH in kidney. # P<0.05 compared with Adult OP, * P<0.05 compared with Adult Ovx; n = 9–11/group.

Three enzymes are reported as responsible for the majority of EETs synthesis - Cyp2J2, Cyp2C2, and Cyp2C6 (rat equivalent to human enzymes 2J2, 2C8 and 2C9). Soluble epoxide hydrolase (sEH) metabolizes EETs to DHETs and is the principal route of EETs metabolism. The major sources of EETs are the liver and kidney [10]. As seen in Figure 3A, liver levels of Cyp2C2 and Cyp2J2 decreased in the Aged OP group, with Cyp2C2 significantly less (p<0.05) than all other groups, and Cyp2J2 significantly less (p<0.05) compared to Adult OP. In contrast, Cyp2C6 was increased significantly (p<0.05) in both Aged groups compared to Adult OP. The sEH levels in the liver were unchanged with estrogen (E2) treatment or age (Fig. 3B). In contrast to the liver, renal Cyp2C2 and Cyp2J2 levels were unaltered with age or E2 (Fig. 3C), while Cyp2C6 was elevated Adult Ovx and Aged OP compared to Adult OP (p<0.05). The sEH levels increased in both E2 treated groups compared to Adult Ovx (Figure 3D; p<0.05).

**Figure 4:**
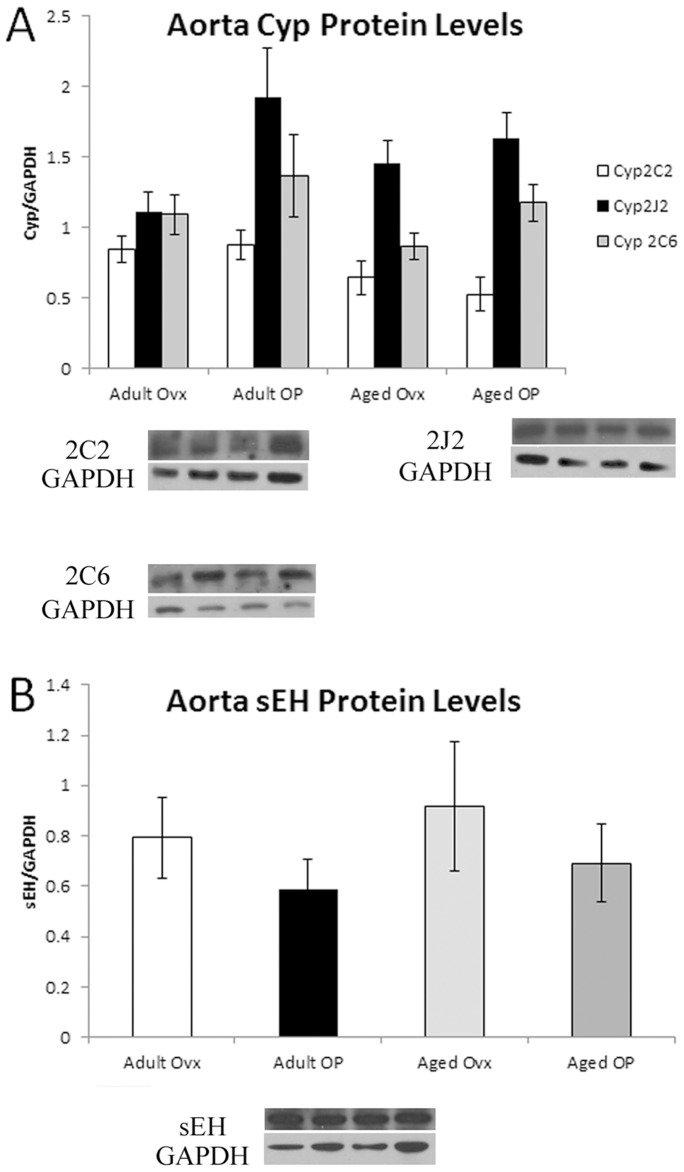
Western blot analysis of aorta EETs related genes, normalized to GAPDH. A representative Western blot showing bands from each of the 4 groups in the order they are given on the graph plus a representative GAPDH blot are shown for each graph. A: Cyp2C2, Cyp2J2 and Cyp2C6 in aorta. B: sEH in aorta. n = 6–9/group.

**Figure 5:**
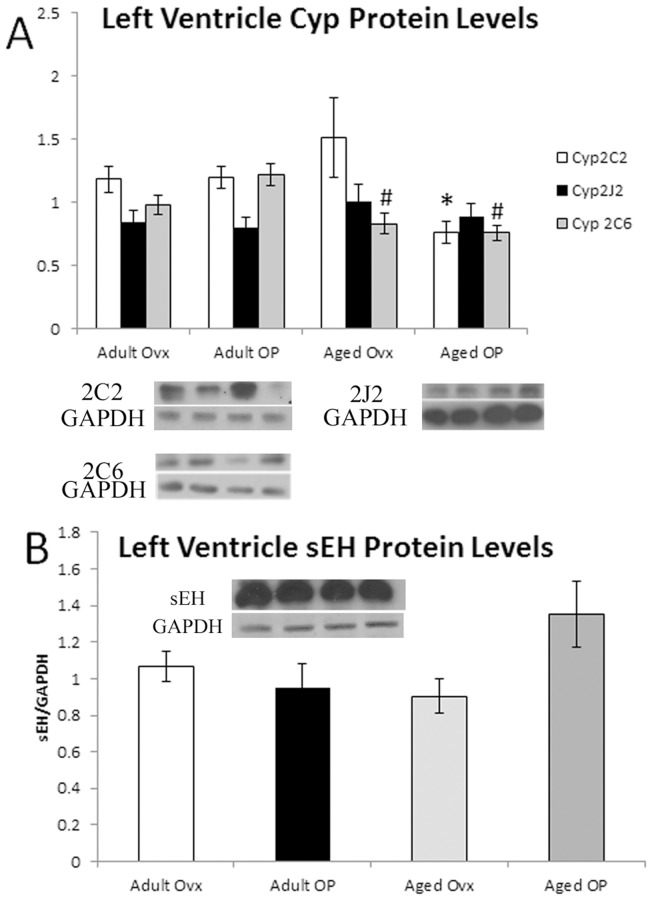
Western blot analysis of left ventricle EETs related genes, normalized to GAPDH. A representative Western blot showing bands from each of the 4 groups in the order they are given on the graph plus a representative GAPDH blot are shown for each graph. A: Cyp2C2, Cyp2J2 and Cyp2C6 in LV. B: sEH in LV. # P<0.05 compared with Adult OP, * P<0.05 compared with Adult Ovx; n = 9–11/group.

**Figure 6:**
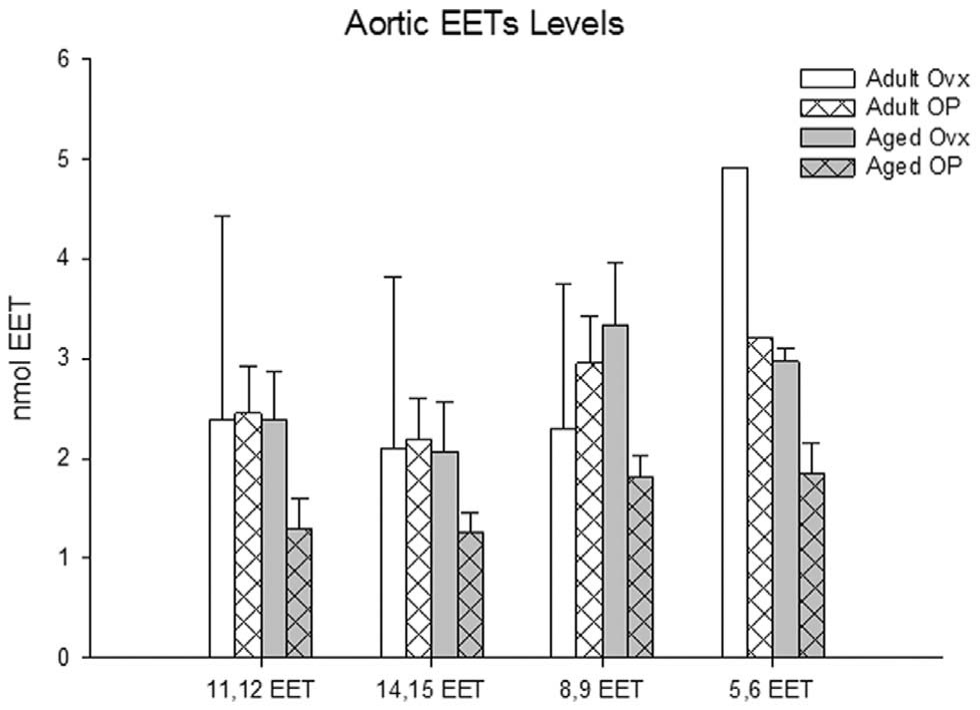
Aortic levels of EETs from MS analysis. Adult Ovx- white bars, Adult OP- white bars with pattern, Aged Ovx- grey bars, Aged OP- grey bars with pattern. n = 3–5/group.

Significant vascular changes occur with aging and estrogen loss include increased stiffness, impaired relaxation and increased atherosclerosis [26], [27]. Aortic levels of Cyp2C2, Cyp2C6 and Cyp2J2 did not vary with age or E2 treatment (Fig. 4A), and the levels of sEH in the aorta were similarly constant (Fig. 4B). In the left ventricle, Cyp2C6 decreased in both Aged groups compared to Adult OP (p <0.05), and Cyp2C2 protein levels decreased in the Aged OP groups compared to Adult Ovx. Cyp2J2 and sEH levels in the left ventricle were unchanged (Fig. 5). Because tissue levels of substances can differ markedly from the plasma levels, we investigated aortic levels of EETs. Tissue levels of EETs did not vary with estrogen or aging for any of the isoforms (Fig. 6).

## Discussion

Levels of Cyp epoxygenases varied in some tissues with aging and estrogen status. Generally, aging led to a decrease in Cyp protein levels, though an increase was seen in liver Cyp 2C6 level. sEH levels, by contrast, varied only in the kidney, based on estrogen status, with both OP groups showing increased amounts of sEH. The measured plasma levels of EETs, however, did not vary among groups.

### EETs Synthesis

EETs are formed by the metabolism of arachidonic acid by cytochrome P450 epoxygenases. In humans, the major Cyps reported to be responsible for EET synthesis are 2C8, 2C9 and 2J2, corresponding to Cyps 2C2, 2C6 and 2J2 in the rat [28], [29]. The principal sources of circulating EETs are reported to be the liver and kidneys [10]. In the liver, aging led to decreased expression of Cyp2C2 and 2J2 and an increase in Cyp2C6. This increase in Cyp2C6 was mirrored in the kidney. EET levels are determined not just by production via the Cyp's, but also by metabolism to DHETs by sEH. Aging had no effect on sEH levels in the liver or kidney; estrogen replacement, however, lead to increased levels of sEH compared to Adult Ovx, as expected from murine studies [30]. Predicting how plasma levels of EETs should vary based on protein expression is complicated by this variation in Cyp and sEH levels. However, the measured plasma levels showed no significant differences in any of the four isomers with either aging or estrogen status. It seems reasonable that there is no change in total EETs given the various increases and decreases in protein levels. It is interesting however, that there is no significant change in the distribution of EET isomers to go along with these changes, given that the different Cyp enzymes can form all of the isomers, but have some level of selectivity [14], [31]. Given that EETs levels do not vary significantly with age or estrogen status, levels of DHETs should largely be dependent principally upon sEH metabolism of the corresponding EETs. The only differences seen were an increase in 14,15 DHET in aged ovx and an increase in 5,6 DHET in adult OP. The only groups with increased sEH expression were adult and aged OP.

The oxylipins measured are free fatty acid oxylipins which have been widely used as biomarkers of biologically active fatty acid chemical mediators [32]–[35]. Given that over 90% of the plasma EETs was esterified to the phospholipids of circulating lipoproteins, it is expected that the downstream effect of these plasma metabolites will be influenced by shifts in lipoprotein metabolism [35], [35]–[37]. The plasma oxylipin levels may not reflect the levels of oxylipins in different tissues, in different plasma fractions, or the oxylipins sequestered as phospholipids and other esters; however, free EETs are thought to be the major biologically active form, with phospholipid EETs being a storage or inactive form. There is little information concerning whether the EETs and other eicosanoids in the phospholipids represent the major pools and generally what conditions mobilize the EETs. In multiple previous studies, the correlation of plasma lipid mediators with biologic activities has proven accurate [32]–[35].

*Plasma EETs levels* - were generally highest in the aged ovx, but this did not reach significance. In contrast, 14,15 DHET levels were clearly increased in aged ovx. Our average values for the various EET isoforms among the groups was 2–8 nM. This is not inconsistent with other groups, however published values vary significantly. Reported levels of 14,15 EET range from 0.3 to 13 nM (human), and 11,12 EET from 0.3 to 48.5 nM (mouse and rat) [38]–[43]. Partly, this is due to differences in methodology. It can be challenging to compare levels between and among papers, because measurements often differ, for example measuring total EETs, versus just 11,12 EET, or 14,15 EET. In addition, nearly all previous studies examined only juvenile or young adult models.

With an interest in EETs as a possible therapeutic for decreasing inflammation, we looked also at Cyp expression in the heart (left ventricle) and vasculature (aorta). In the heart, Cyp 2C6 was decreased in both aged groups. Cyp 2C2 was decreased in the Aged OP group, while Cyp 2J2 was unchanged. Neither aging nor estrogen status effected sEH. The changes in Cyp and sEH protein expression are similar to that in the liver. Polymorphisms in Cyp2J2 are associated with increased risk of coronary artery disease [44]. The polymorphism investigated in this case led to a decrease in measured plasma DHETs levels. While the decrease in Cyp2J2 in the liver measured with aging in our study did not have a corresponding decrease in measured EETs or DHETs, this relative loss of Cyp2J2 may contribute to an increased risk of CAD and related diseases.

In contrast, no significant differences in any of the measured proteins were detected in the aorta. Vascular (aortic tissue) levels of EETs were measured, and, similar to plasma levels, found to have no significant differences based on aging or estrogen status. These levels reflect the lack of difference in protein expression among groups.

Overall EETs are considered anti-inflammatory and beneficial. EETs are anti-inflammatory and promote angiogenesis [14]. However there may be a downside to this, as a recent study using adenovirus mediated overexpression of (human) Cyp 2C8 and Cyp 2J2, along with an sEH null, in mice, found increased tumor metastasis [45]. The increased levels of EETs in these mice also promoted tumor growth. Measured levels of EETs were approximately 0.3 nM in the Cyp overexpressed mice and 4.2 nM for the sEH null mice, in a similar range to EETs levels reported in the literature and this study, as discussed above.

### EETs and Cardiovascular Protection

Most *in vivo* studies of EETs and cardiovascular protection have used sEH inhibitors to increase EETs levels, given the short half-life of EETs. Two studies involving vascular damage showed benefit: one study showed decreased inflammatory markers, rate of abdominal aortic aneurysm formation and atherosclerotic lesion area, while another showed a decrease in neointima formation as well as a decrease in smooth muscle proliferation and expression of pro-inflammatory genes in a femoral cuff model [46], [47]. However, there was neither improved outcome with sEHi n following carotid artery ligation, nor decreased macrophage adhesion with the femoral cuff model [46], [47]. EETs or sEHi treatment have a been found to reduce infarct size and contribute to preconditioning [48], [49]. EETs also have been shown to reduce apoptosis in neonatal rat cardiac myocytes after hypoxia/reoxygenation [50]. Interestingly, sEHi prevented stroke in spontaneously hypertensive stroke-prone rats without reducing blood pressure [51]. Thus EETs or sEHi have protective properties in the cardiovascular system.

Knowledge of the normal levels of EETs in organisms can give perspective on observed effects of EETs' treatments in cell culture as well as *in vivo*. There is great interest in EETs anti-inflammatory and anti-apoptotic properties in cell and tissue injury. Dhanasekaran et al. found 1 uM of 8,9-, 11,12-, or 13–15-EETs reduced apoptosis after hypoxia/reoxygenation in HL-1 cells and in neonatal cardiac myocytes [50]. EETs activated the pro-survival pathway, PI3K/Akt. In another study, it was observed that 1 uM 11,12-EET increased sphingosine kinase-1(SK1) activity by 110% [52]. 11,12-EETs also increased endothelial cell proliferation, and this was prevented by selective inhibition of SK1 [52]. Lipopolysaccharide (LPS) induces a strong pro-inflammatory response including the production of inflammatory prostaglandins. In monocytes, 10 uM 11,12 EET prevented increased PGE_2_ synthesis after LPS treatment [53]. Concentrations of EETs in this set of studies ranges from 1 uM to 10 uM. An important question is whether the observed protective effects of EETs in these studies represent basal activity or pharmacologic. In the current study we found that plasma EETs levels are less than 10 nM for each of the four EETs. Thus the observed protective effects in these studies occurred with pharmacologic levels of EETs, and may not occur with physiologic concentrations.

### Arachidonic Acid Metabolism, Aging and Estrogen Loss

In the current study, EETs metabolism varied little with estrogen loss and aging. In contrast, the cyclooxygenase pathway, which can lead to the production of ROS as well as prostaglandins and via COX-1 and 2 , has been found to change with aging and estrogen loss [54]. Aging alone was found decrease arteriole expression of COX-1, but not to affect COX-2 expression [55]. Estrogen blocked increased expression of COX-2 in the vasculature and inhibited increased production of PGE2 in response to LPS and other stimuli [56], [57]. This protective effect was lost with aging. Similarly COX derived prostanoids and ROS increase with aging and estrogen loss, contributing to vascular dysfunction [54]. Studies on the lipoxygenase (LO) metabolites of arachidonic acid in aging and estrogen loss are very limited. In a single study, pulmonary artery contractions were much greater in female than male rabbits in response to LO arachidonic acid metabolites, and this response was secondary to increased levels of 15-Hete [58]. This correlated with increased expression of 15-LO in female rabbit arteries. Thus, overall there is an increase in pro-inflammatory responses in the prostaglandin arm of arachidonic acid metabolism with aging and loss of estrogen. In contrast, in the current study EETs metabolism showed little variation with aging and estrogen loss.

### Limitations

In our studies we used standard rat chow, which often contains significant amounts of soy protein and phytoestrogens. It is possible that phytoestrogens's estrogen-like properties may have masked differences, even though our approach results in marked differences in E2 levels amongst the groups and we have consistently found differences in other studies using the same model [26], [59], [60]. Serum samples were collected three different times and there was variability amongst serum samples from the same group (e.g. adult ovx) of rats housed together, eating the same rat chow. Therefore, we think it unlikely that phytoestrogens masked differences, but cannot completely exclude this possibility. Some clear differences have been found with phytoestrogens in a number of studies including a decrease in atherosclerosis in the Apo E knockout mouse, decreased soluble vascular cell adhesion protein (VCAM)-1, increased eNOS expression, increased antioxidants and decreased monocyte chemo-attractant protein (MCP) -1 [61]–[64]. In another study, MCP-1 did not change with a soy supplemented diet [63]5611. Other studies with different endpoints have found no or very limited phytoestrogen effects [65], [66]. In fact, many excellent labs have used standard rat chow in their studies, again finding significant differences with changes in estrogen [67]–[70]. Nonetheless, we cannot completely exclude phytoestrogens in standard rat chow as contributing to the variability in EETs levels, which is most apparent in the aged ovx group.

We have investigated the major enzymes responsible for circulating and cardiac levels of EETs and DHETs. While there is no significant change in EETs levels with either aging or estrogen status, there are some changes in Cyp protein expression. Increased inflammation seen with aging, then, is not due to a loss of circulating EETs and at least this arm of the arachidonic acid metabolism pathway is preserved. While this means that the inflammatory changes of aging and estrogen loss cannot be decreased by replacement of lost EETs, they may nonetheless be counterbalanced by increasing EETs levels beyond those naturally produced, either with EETs supplementation or sEHi administration. Further investigation of the *in vivo* effects of elevated EETs levels are needed.
